# Improving Patient Choice, Autonomy and Relational Security Through a Co-Produced ‘My Story’ Booklet on Saunders Ward–Low Secure Unit: A Quality Improvement Project

**DOI:** 10.1192/bjo.2026.11253

**Published:** 2026-06-30

**Authors:** Malik Awan, Sukhwinder Kaur, Zoe Butler, Jane Ngwenya, Caitlyn Price

**Affiliations:** Cygnet Healthcare, Stevenage, United Kingdom

## Abstract

**Aims:**

The Independent Review of the Mental Health Act (MHA) (2018) by Sir Simon Wessely and subsequent MHA White Paper (2021) recommended promoting patient choice, autonomy and least restrictive practice and suggested quality improvement (QI) programme. NHS England led National MHA QI Programme (July 2024– March 2025) across 52 trusts including Saunders ward, Cygnet Stevenage. The aim was to enhance patients’ reported sense of autonomy, strengthen patient voice, and improve relational engagement with staff by March 2025 through co-production and implementation of an individualised “My Story” booklet.

**Methods:**

The project was supported by an NHSE QI Coach and Cygnet Executives. We used an evidence-based change approach to identify a local quality gap and targeted improvement ideas. Baseline information was gathered through equity huddle discussions, apre-QI patient questionnaire, and the *Fearless Organisation* survey to explore staff psychological safety. The key areas of inequity identified: underrepresentation of patient’s views, lack of support to identify strengths, and communication gaps with the staff especially non-permanent staff.

To address these, PDSA cycle was used to co-produce a patient-led, individualised “My Story” booklet. Five patients participated in the pilot, creating booklets describing their interests, personal journeys and future goals. The booklets were confidentially stored and made accessible to all staff to support personalised and relationally informed interactions. By making patients’ identities and preferences visible, the intervention was intended to enable more personalised interactions, thereby supporting relational engagement and patient autonomy.

**Results:**

Following implementation of “My Story” booklet, patients reported improved relational engagement with staff during routine day-to-day interactions. Post-intervention questionnaires demonstrated improvement across targeted domains, ability to express preferences and interests to staff (60%). Qualitative feedback indicated that staff, including agency staff, initiated more personalised conversations, such as enquiring about patients’ activities or future goals. Given the small pilot sample (n=5) limited statistical significance, patient testimonies reflected increased confidence in engaging with staff and a desire for continued involvement and further development of their “My Story” booklet.

**Conclusion:**

This co-produced QI intervention supported aims of MHA reform by enhancing patient autonomy and relational engagement within a low secure setting. By making patients’ identities and preferences visible to all staff, the intervention facilitated more personalisedand meaningful interactions. As a small-scale pilot, the project demonstrates potential value of simple, patient-led tools in strengthening relational security and supporting less restrictive, person-centred care. Future work will focus on embedding the intervention into routine practice and exploring its applicability across other secure inpatient settings.

